# Impact of liquidity spillovers among industrial sectors on stock markets during crisis periods: Evidence from the S&P 500 index

**DOI:** 10.1371/journal.pone.0277261

**Published:** 2022-11-17

**Authors:** Seo-Yeon Lim, Sun-Yong Choi

**Affiliations:** 1 Department of Economics, Gachon University, Seongnam, Republic of Korea; 2 Department of Financial Mathematics, Gachon University, Seongnam, Republic of Korea; Massey University - Albany Campus: Massey University - Auckland Campus, NEW ZEALAND

## Abstract

We investigate liquidity spillovers among industry sectors in the S&P 500 index to explain the interconnection dynamics in the US stock market. To do so, we define a sectoral liquidity measure based on the Amihud liquidity measure. Employing the spillover model, we further examine US sectors’ liquidity spillovers during the global financial crisis (GFC) and the COVID-19 pandemic. Based on the relationship between liquidity in financial markets and business cycles, our findings show that (i) liquidity connections became stronger during both crises, (ii) in the GFC period, the material sector was the primary transmitter of total liquidity spillovers, whereas in the COVID-19 pandemic period, the consumer discretionary sector was the main conveyor of total liquidity spillovers and the real estate sector was the dominant recipient of total liquidity spillovers, and (iii) net liquidity spillovers between all sectors fluctuated notably during the GFC, while the industrial, consumer staples, and healthcare sectors had the largest net liquidity spillovers during the COVID-19 crisis. These findings have important implications for portfolio managers and policymakers.

## Introduction

Asset managers and investors acknowledge the importance of liquidity in the financial market in terms of the relationship between liquidity and the return on their investments, as well as the role of liquidity in a portfolio evaluation ([1]). In other words, liquidity affects actual returns because illiquid assets cost more to trade. Consequently, they eat into an investor’s returns. Additionally, the evaluation of a portfolio is influenced by liquidity, as more illiquidity devalues the portfolio. Therefore, market participants should pay attention to liquidity to comprehend risk management, asset pricing, and portfolio evaluation. Recognizing the importance of liquidity, several scholars have studied market liquidity to further offer crucial insights.

Research on liquidity mainly focuses on the stock market. First, the research on the relationship between stock prices and liquidity is ample ([2–10]). Second, some studies have examined liquidity spillovers between cross-markets worldwide ([11–14]). However, the research on liquidity spillovers based on sectoral data is scant ([15, 16]).

Various measures have been introduced to calculate liquidity. In accordance with [17], liquid stocks are defined as stocks that can trade large volumes quickly at low cost with little price impact. From this definition, [18] distinguished between four dimensions of stock liquidity: trading quantity, trading speed, trading costs, and price impact. Regarding trading quantity, there are several volume-based measures that consider the number of transactions. For example, the trading volume is a straightforward proxy for liquidity. Another volume-based measure is turnover ratio, which is the number of traded shares divided by the number of outstanding shares. Regarding trading costs, the bid/ask spread is considered one of the best transaction-cost-based measures. There are many proxies for bid-ask spreads, including [19] implicit effective spread, Zeros ([20]), high-low ratio spread estimator ([21]), closing percent quoted spread ([22]), an estimator that improves the roll measure ([23]), and the high-low range measure ([24]). Further, the [5] liquidity measure is one of the most commonly used price-impact-based measures. This is the illiquidity measure (ILLIQ), the daily ratio of absolute stock return to its dollar volume averaged over a given period. Some measures are also based on the illiquidity ratio: volatility over volume ([25]) and the open-to-close Amihud measure ([26]). The return-to-turnover ratio ([27]) is also an example of price-based measures. Lastly, there is a well-known multidimension-based measure: the turnover-adjusted number of zero daily trading volumes ([17]). This measure considers multiple dimensions of liquidity, including trading quantity, trading speed, and trading cost, with a particular focal point on trading speed.

The COVID-19 pandemic has affected the global economy in terms of both supply and demand ([28–31]). Moreover, COVID-19 has also notably influenced financial markets worldwide, including in China, Japan, and European countries ([32, 33]). In particular, the COVID-19 pandemic has notably affected the US financial market and individual firms ([34–37]). Similarly, the global financial crisis (GFC), which originated in the real estate sector, has had major impacts on the global economy ([38, 39]). Furthermore, the GFC has significantly influenced financial markets in many regions, such as China, India, and European countries ([40–44]). Specifically, the GFC has created financial linkages across international stock markets ([45–48]). Since the GFC and COVID-19 pandemic have caused enormous damage to the global economy, some scholars have conducted comparative studies on the impact of crises on the financial market during the GFC and COVID-19 periods ([49–52]).

This study investigates the spillover effects of sectoral liquidity. In other words, we define the sectoral liquidity measure and examine the relationship of liquidity among the 11 sectors in the S&P 500 Index. We use [5]’s liquidity measure to estimate liquidity in all sectors. The Amihud measure has three main advantages over other liquidity measures, pursuant to [53]. First, the Amihud measure was constructed using the absolute value of the daily return-to-volume ratio to predict the price impact. Second, the measure collects daily data and therefore provides a longer time series, unlike proxies based on intraday data. Third, a strong positive relationship is observed between measured and expected stock returns ([5, 54], among many other studies). Admitting these advantages, many scholars employ the Amihud liquidity measure ([55–58]). We also analyze individual industries rather than the entire market, because investigating entire markets may entail bias and conceal useful information about the behavior of individual industries ([59–62]). Furthermore, we include the GFC and COVID-19 pandemic periods in the sample data to analyze how the relationship differs statically and dynamically during these two crisis periods.

We employ spillover analysis introduced by [63] to evaluate the dynamic interdependence of liquidity between sectors. [63] introduced a volatility spillover model based on forecast error variance decompositions (FEVD) from a vector autoregression (VAR) framework. The model complements the methodological limitations of [64]. [63] developed a VAR framework in which FEVD is invariant to variable ordering and explicitly derives directional volatility spillovers. According to the spillover index, we calculate liquidity spillovers among the 11 sectors in the S&P 500 index. Furthermore, we conduct static spillover and rolling-window analyses to discover dynamic linkages of liquidity among sectors. Many studies employ spillover analysis to investigate the dynamic relationship between financial assets ([65–70]). However, to the best of our knowledge, no study has examined the interconnection of liquidity between sectors.

Conventional beliefs suggest that market liquidity differs depending on economic conditions. Asset liquidity and liquidity interconnectedness among assets vary with business cycles. Unexpected liquidity transmissions concur with economic crises ([71–75]). Thus, acknowledging the interrelationship between financial liquidity and the real economy, we conduct liquidity spillover analysis in this study. Furthermore, we theoretically explain the liquidity co-movement among sectors based on herding behavior. In financial markets, herding behavior refers to the process by which market participants deduce information from the behavior of previous participants ([76]). Therefore, some studies cite herding behavior to explore the spillover effects in financial markets ([77–79]). Based on this background, our primary findings are as follows: First, the total liquidity spillovers during the GFC and COVID-19 periods were higher than those in the full period, proving that sectors were more connected in liquidity aspects during the crises. Second, the material sector was the primary transmitter of total liquidity spillovers during the GFC. In contrast, during the COVID-19 pandemic, the consumer discretionary sector was the main conveyor of total liquidity spillovers, and the real estate sector was the dominant recipient of total liquidity spillovers. Third, net liquidity spillovers in all sectors fluctuated significantly during the GFC, while they showed remarkable changes during the COVID-19 crisis, with particular sectors such as industrials, consumer staples, and healthcare having the largest net liquidity spillovers.

This study contributes to financial literature in several ways. First, the analysis of liquidity interlinkages among the 11 sectors is a very interesting topic for market participants, as the analysis is the first to introduce a liquidity measure. Through this, it helps to understand changes in the relationship between industries in a liquidity perspective. Second, we examine the dynamic relationship of liquidity between sectors during the two crisis periods. We notice the primary findings with respect to total and net liquidity spillovers. Our findings suggest that sectors are closely related through the channel of liquidity transmission during crises. Third, we use the spillover analysis introduced by [63], but focus on liquidity. To the best of our knowledge, previous studies have investigated spillovers from different perspectives ([80–82]). This is the first study to employ it in liquidity aspects; therefore, it is novel to scrutinize the interconnection of liquidity among sectors. Lastly, our findings have useful implications for portfolio managers, investors, and policymakers. By shedding light on the liquidity spillovers of various sectors in the US stock market, all the participants related to the stock market can consider these results and consequently make better decisions.

This paper proceeds as follows. Liquidity measure and methodology present the sectoral liquidity spillover measurement based on the Amihud liquidity measure and [63]’s volatility spillover model. Empirical results present the results of the study. Concluding remarks summarize the findings and conclude the study.

## Literature review

### Stock price and liquidity

Numerous studies have examined the link between liquidity and expected stock returns. Some studies investigated this relationship by defining liquidity as the bid/ask spread ([2, 83, 84]). To start with, [2] showed that the market-observed expected return is an increasing and concave function of the bid/ask spread. Similarly, [83] noted that liquidity is an important factor in stock returns, representing the bid/ask spread as a liquidity measure. More specifically, they investigated the effects of illiquidity on the yields of finite-maturity securities with identical cash flows: U.S. treasury bills and notes with maturities under six months. Moreover, [84] examined the relationship between average returns and bid-ask spreads, particularly in the January and non-January months. Using NYSE monthly stock returns over the 1961–1990 period, they showed that the liquidity premium is reliably positive only in January.

One study investigated the return-illiquidity relationship by employing a different liquidity measure. [85] estimated measures of illiquidity from intraday transactions data and use the [3] factors to adjust for risk. Their main findings prove that the relationship between a return premium and a variable cost is concave, while a convex relationship between the premium and the estimated fixed cost is observed.

Other studies have used volume-based measures to estimate liquidity ([4, 5, 86, 87]). First, [4] proposed the turnover rate of an asset as a proxy for its liquidity. They found that there is a significantly negative relationship between stock returns and their turnover rates, confirming that illiquidity stocks yield higher average returns. Furthermore, [86] estimated liquidity using two measures of trading activity: the dollar trading volume and share turnover. By documenting negative and significant cross-sectional relationships between average stock returns and measures of trading activity, such as dollar volume and share turnover, they showed that variables related to trading activity play an important role in the cross section of expected returns. [5] employed volume-based illiquidity measures referred to as ILLIQ, which is the daily ratio of absolute stock return to its dollar volume, averaged over a given period. Unlike other researchers, he examined the data not only across stocks but also over time using NYSE stocks during the period from 1994 through 1997. Additionally, there is research on the link between sensitivity to liquidity and the average return on stocks. [87] found that stocks that are more sensitive to aggregate liquidity have substantially higher expected returns, even after accounting for exposures to the market return as well as size, value, and momentum factors.

Several studies have scrutinized the relationship between shocks to liquidity and changes in stock returns ([6, 88]). First, [88] derived a liquidity-adjusted Capital Asset Pricing Model (CAPM) to show the link between a security’s required return and its liquidity using NYSE and AMEX stocks during the period from 1963 through 1999. They showed how a persistent negative shock to a security’s liquidity affects both contemporaneous and predicted future returns. Similarly, [6] examined the change in expected returns caused by systematic variations in liquidity in emerging equity markets, where liquidity ought to be particularly important. They found that unexpected liquidity shocks are positively correlated with contemporaneous return shocks and negatively correlated with dividend yield shocks.

Many scholars have explored topics pertaining to liquidity ([7, 89–92]). To exemplify, [89] found commonality across assets for each individual measure of liquidity and that these common factors are correlated across measures of liquidity. [90] further showed that greater liquidity results in higher informational market efficiency. Finally, [7] suggested some key liquidity drivers, namely liquidity shocks in the effective spread, the number of trades, and the arrival of firm-specific rather than macroeconomic news.

Recently, further studies on stock prices and liquidity have been conducted ([8–10]). Initially, based on the view that the accumulation of bad news is the key factor in the formation of a stock price crash, [8] examined the relationship between stock liquidity and stock price crash risk. These results suggest that high stock liquidity can increase managers’ incentives to withhold bad news and weaken financial market stability. Likewise, [9] investigated the route through which illiquidity systematic risk has affected expected stock returns. They find that expected returns are higher for stocks with higher sensitivity to the illiquidity return premium factor: the premium on illiquid-minus-liquid stock portfolios (IML) ([93, 94]) in times of greater financial distress and funding illiquidity. Additionally, [10] introduced a novel two-factor model. This model includes market and liquidity factors to explain the significant effect of liquidity on stock returns in the Chinese markets, which neither the CAPM nor the Fama-French models can take into consideration.

### Spillover analysis on liquidity

A number of studies have investigated the spillover analysis of liquidity across international financial markets ([11–13, 95, 96]). To begin with, using data from the U.S., British, Brazilian, and Hong Kong stock markets, [11] attempted to verify the scale correlation between their illiquidities from June 2005 to September 2012, to consider the non-crisis, sub-prime crisis, and Eurozone crisis. They empirically confirmed that there are variations in the magnitude of liquidity spillovers in international markets depending on the crisis period. Further, using data from the G7 stock markets, [95] examined the dynamics of illiquidity spillovers across countries. They finally concluded that overall, the interdependence of illiquidity, returns, and risk is structurally unchanged during international financial crises. Third, [12] empirically investigated illiquidity and volatility spillover effects in eight developed equity markets during and after the global financial crisis. [12] suggested a significant intercorrelation between illiquidity and volatility across borders and further proposed a multiplicative error model (MEM) for the dynamics of illiquidity and volatility. However, they also noted that the causal effects between illiquidity and volatility in their own markets are trivial. Furthermore, [96] investigated the cross-market link of liquidity spillovers between nine Asian and five developed stock markets from 2006 to 2016. Their findings suggest bidirectional causality in market liquidity among not only developed markets, but also Asian emerging markets. They also noted that regional spillovers in Asian markets are larger than those in developed markets. In conclusion, [13] found a relation between liquidity spillovers and GDP synchronization across 24 countries during the period from 1990 to 2018. Ultimately, they perceived that liquidity spillovers could generate an output divergence.

Liquidity spillover analysis has been applied to non-stock financial assets ([14, 97]). [97] observed the magnitude of liquidity spillover between the ETF and its underlying assets. Using daily data of the DIAMONDS ETF on the Dow Jones Industrial Average and its underlying stocks from April 2002 to December 2016, their results indicate that the liquidity spillover between them is significant, especially during the market crisis. In contrast, [14] explored liquidity spillovers across nine major foreign exchange (FX) markets during the period from 2008 to 2015. They indicated that FX liquidity spillover is time dependent and significantly higher during the US subprime mortgage and European sovereign debt crises.

Some studies have conducted spillover analyses on sectoral data ([15, 16]). First, [15] captured liquidity interlinkages among U.S. industrial sectors using S&P 500 firms from January 1, 2012, to July 17, 2020. They analyzed how COVID-19 has affected liquidity interconnectedness, concluding that this interrelationship has grown significantly from the pre-COVID to COVID periods. Specifically, the pandemic has an uneven effect, with the utility sector being the most affected and the telecommunication services sector the least. Similarly, [16] investigated price co-movement among the four families of NASDAQ commodities (energy, agriculture, precious metals, and industrial metals). Finally, they observe a substantial positive correlation between price co-movements across different commodity markets. This comovement is driven by the cross-sectional liquidity spillover effect.

## Liquidity measure and methodology

### Liquidity measure

We employ Amihud’s measure to evaluate the liquidity for each section ([5]). It is based on the intuition of a security’s price impact and is advantageous in that it can be easily calculated from daily stock price and volume data ([98–100]).

The Amihud liquidity measure is defined as follows:

$$\begin{array}{c} L_{i,t}=\frac{1}{N}\sum_{j}^{N}\frac{|R_{j,t}|}{V_{j,t}}, \end{array}$$ (1)

where *N* is the total number of stocks in a sector at time *t* and *R*_*j*,*t*_ is the daily return of *j*-th stock in the sector at time *t*. The total number of shares (*N*) changes as the incorporated stock changes for a sector. *V*_*j*,*t*_ is the dollar trading volume of the *j*th stock at time *t*. Therefore, we refer the liquidity measure as the average sector liquidity measure.

Specifically, we obtain daily closing prices and volumes of all stocks that make up the S&P 500 index from January 2004 to March 2022 from finance.yahoo.com. Volume measures the number of a stock’s shares that are traded on a stock exchange in a day. We then calculate the dollar trading volume for each stock by multiplying the closing price by the volume of the stock. In addition, the sample data period includes crisis periods such as the 2008 GFC, European debt crisis (EDC), and COVID-19 pandemic. Based on the sample data, we calculate the average liquidity for each sector using Eq (1).

Table 1 lists the summary statistics for liquidity in all sectors during the entire period from January 2004 to March 2022. On average, MAT had the highest liquidity (1.7161) and ENG had the lowest (0.0116). Based on the standard deviation, the liquidity of MAT varied significantly, reaching 11.3702, whereas that of ENG and UTI varied slightly, at 0.0118 and 0.0164, respectively. Skewness indicates the asymmetry or distortion of a distribution. All sectors display positive skewness, which means that most values were clustered around the left tails, while the right tails of the distributions were longer. In particular, the highest skewness was observed in CS (41.74) and FIN (53.61), whereas those of RE and UTI were the lowest, accounting for 3.13 and 3.04 each. The kurtosis determines the heaviness of the distribution tails. FIN had the highest kurtosis value at 3250.04, suggesting that the distribution of FIN’s liquidity tends to have the heaviest tails. In contrast, the lowest value was observed for RE at 15.42. According to the Jarque-Bera test, the null hypothesis of normally distributed average liquidity is rejected for all sectors.

**Table 1:** Summary statistics for the average sector liquidity measure for the 11 sectors in the S&P 500 index. The Jarque-Bera statistic tests the null hypothesis of normality for the sample returns.

Sectors	Mean	Max.	Min.	Std. dev.	Skewness	Kurtosis	Jarque-Bera
Communication Services (CS)	0.0424	19.566	0.0021	0.3582	41.74	2072.74	82227.44 × 10^4^ ^‡^
Consumer Discretionary (CD)	0.0522	14.7222	0.0026	0.2767	37.02	1792.45	61498 × 10^4^ ^‡^
Consumer Staples (CST)	0.0199	2.3737	0.0016	0.0831	18.44	423.86	3459.01 × 10^4^ ^‡^
Energy (ENG)	0.0116	0.1784	0.0011	0.0118	4.15	30.52	19.12 × 10^4^ ^‡^
Financials (FIN)	0.0781	47.6478	0.0021	0.7671	53.61	3250.04	202056.99 × 10^4^ ^‡^
Healthcare (HC)	0.0668	1.2208	0.0019	0.0915	3.26	18.02	7.02 × 10^4^ ^‡^
Industrials (IND)	0.0393	3.0029	0.0027	0.0608	27.19	1250.56	29940.12 × 10^4^ ^‡^
Information Technology (IT)	0.0607	1.2523	0.0023	0.0723	5.01	44.71	40.1 × 10^4^ ^‡^
Materials (MAT)	1.7161	213.4401	0.0019	11.3702	10.37	130.57	334 × 10^4^ ^‡^
Real Estate (RE)	0.0389	0.6795	0.0021	0.0532	3.13	15.42	5.29 × 10^4^ ^‡^
Utilities (UTI)	0.018	0.2327	0.0016	0.0164	3.04	18.27	7.09 × 10^4^ ^‡^

^‡^ indicates a rejection of the null hypothesis at the 1% significance level.

### The spillover index

To investigate the liquidity spillover effect among sectors in the US stock market, we employ the spillover index approach developed by [63] (hereafter, DY). This method is based on forecast error variance decompositions (FEVD) from a vector autoregression (VAR) model ([101, 102]).

We briefly introduce an approach based on previous studies ([63, 65–68, 103]). This approach begins with a covariance stationary *N*-variable VAR model with lag *p* as follows:

$$\begin{array}{c} X_{t}=\sum_{i=1}^{p}\phi_{i}X_{t-i}+\epsilon_{t}, \end{array}$$ (2)

where *X*_*t*_ indicates the *N*-dimensional column vector of the variables at time *t* and *φ*_*i*_ is the *N* × *N* parameter matrix. *ϵ*_*t*_ ∼ (0, Σ) is *N* × 1 error vector and independent and identically distributed.

As *X*_*t*_ is a covariance stationary process, the VAR process in Eq (2) can be expressed as follows:

$$\begin{array}{c} X_{t}=\sum_{i=0}^{\infty }A_{i}\epsilon_{t-i}, \end{array}$$ (3)

where *N* × *N* coefficient matrices *A*_*i*_ have the following recursive pattern:

$$\begin{array}{c} A_{i}=\phi_{1}A_{i-1}+\phi_{2}A_{i-2}+\cdots +\phi_{p}A_{i-p}, \end{array}$$

where *A*_0_ is an *N* × *N* identity matrix and *A*_*i*_ = 0 for *i* < 0.

Furthermore, based on the generalized VAR framework conceived by [101, 102], the DY model decomposes the forecast error variance of Eq (3). Accordingly, the *H*-step-ahead FEVD from variable *i* to variable *j* can be written as:

$$\begin{array}{c} \theta_{ij}\left(H\right)=\frac{\sigma_{jj}^{-1}\sum_{h=0}^{H-1}{\left(e\prime_{i}A_{h}\sum e_{j}\right)}^{2}}{\sum_{h=0}^{H-1}\left(e\prime_{i}A_{h}\sum A\prime_{h}e_{i}\right)}, \end{array}$$ (4)

where Σ is the variance matrix of the error vector *ϵ*, *σ*_*jj*_ is the standard deviation for the *j*-th equation, and *e*_*i*_ is a selection vector, where one is the *i*-th element, and zero otherwise.

To normalize the sum of each row in Eq (4), we standardize each entry using the row sum as follows:

$$\begin{array}{c} {\tilde{\theta }}_{ij}\left(H\right)=\frac{\theta_{ij}\left(H\right)}{\sum_{j=1}^{N}\theta_{ij}\left(H\right)}, \end{array}$$ (5)

such that $\sum_{j=1}^{N}{\tilde{\theta }}_{ij}\left(H\right)=1$ and $\sum_{i,j=1}^{N}{\tilde{\theta }}_{ij}\left(H\right)=N$.

Following the DY model, we construct the total spillover index(≔ *TSI*(*H*)), as follows:

$$\begin{array}{c} TSI\left(H\right)=\frac{\sum_{i,j=1,i\neq j}^{N}{\tilde{\theta }}_{ij}\left(H\right)}{\sum_{i,j=1}^{N}{\tilde{\theta }}_{ij}\left(H\right)}\times 100=\frac{\sum_{i,j=1,i\neq j}^{N}{\tilde{\theta }}_{ij}\left(H\right)}{N}\times 100. \end{array}$$ (6)

Furthermore, the directional spillovers received by sector *i* from all other sectors *j*(≔ *DS*_*i*⋅_(*H*)) are measured as follows:

$$\begin{array}{c} DS_{i\cdot }\left(H\right)=\frac{\sum_{j=1,j\neq i}^{N}{\tilde{\theta }}_{ij}\left(H\right)}{N}\times 100. \end{array}$$ (7)

Similarly, the directional spillovers transmitted by sector *i* to all other sectors *j*(≔ *DS*_⋅*i*_(*H*)) are estimated as follows:

$$\begin{array}{c} DS_{\cdot i}\left(H\right)=\frac{\sum_{j=1,j\neq i}^{N}{\tilde{\theta }}_{ji}\left(H\right)}{N}\times 100. \end{array}$$ (8)

In addition, the net spillovers from sector *i* to all other sectors *j*(≔ *NS*_*i*_(*H*)) are estimated as

$$\begin{array}{c} NS_{i}\left(H\right)=DS_{\cdot i}\left(H\right)-DS_{i\cdot }\left(H\right). \end{array}$$ (9)

## Empirical results

We analyze liquidity spillovers among 11 sectors (Communication Service (CS), Consumer Discretionary (CD), Consumer Staples (CST), Energy (ENG), Financials (FIN), Health Care (HC), Industrials (IND), Information Technology (IT), Materials (MAT), Real Estates (RE), and Utilities (UTI)) using the S&P 500 index during the period including the GFC and COVID-19. Specifically, through statistical and rolling-window analyses, we compare the liquidity spillovers of each sector and investigate the dynamic liquidity spillovers during the two crises. Based on herding behavior and the relationship between liquidity and business cycles, we further find the implications of our results.

### Statistical analysis

We first estimate the liquidity spillovers among 11 sectors for the entire period. We further divide the full period into two sub-periods namely the GFC (from August 2007 to March 2009) and the COVID-19 pandemic (from January 2020 to March 2022). Through sub-period analysis, we can investigate the liquidity spillovers among the sectors in the US stock market during the two crisis periods, and compare the associated liquidity spillovers.

According to Table 2, the TSI is 22.63%, indicating weak liquidity connections between all sectors. Additionally, HC and IND had the largest (27.74%) and smallest (-17.03%) NETs among all sectors. In other words, HC significantly affects other sectors’ liquidity, while IND is significantly affected by other sectors’ liquidity. As for the directional spillovers between sectors, HC was a main sector transmitting total liquidity spillovers to all other sectors, constituting 68.91%. In contrast, UTI received total liquidity spillovers from all other sectors, with the highest proportion of 53.42%. Moreover, the smallest percentages of 0.30% and 0.81% were observed in CD, as a transmitter and receiver of total liquidity spillovers, respectively.

**Table 2:** Summary of liquidity spillovers in all the sectors for the entire period. The forecast horizon is *H* = 30. The optimal lag order is determined by the AIC. Notes: TSI = total spillover index in (6); FROM = *DS*_*i*⋅_(*H*) in (7), total liquidity spillovers received by the *i*-th sector from all other sectors; TO = *DS*_⋅*i*_(*H*) in (8), total liquidity spillovers transmitted by the *i*-th sector to all other sectors; TO (own) = total liquidity spillovers generated by the *i*-th sector, including the contribution of its own; NET = *NS*_*i*_(*H*) in (9), net spillovers (the difference between transmitted liquidity shocks and received liquidity shocks).

	CS	CD	CST	ENG	FIN	HC	IND	IT	MAT	RE	UTI	FROM
CS	0.9903	0.000	0.0001	0.0002	0.0001	0.003	0.0047	0.0001	0.0002	0.0004	0.0009	0.0097
CD	0.000	0.9919	0.0001	0.0005	0.000	0.0035	0.0005	0.0003	0.0002	0.0011	0.0019	0.0081
CST	0.0003	0.0003	0.8625	0.0191	0.0018	0.0284	0.0036	0.0011	0.0001	0.0574	0.0254	0.1375
ENG	0.0001	0.0002	0.007	0.6818	0.0002	0.0813	0.0148	0.0522	0.0013	0.066	0.0951	0.3182
FIN	0.0001	0.000	0.0026	0.0086	0.9165	0.0113	0.0037	0.0012	0.0001	0.0296	0.0262	0.0835
HC	0.000	0.0008	0.0078	0.0407	0.0013	0.5883	0.0324	0.0323	0.0015	0.1772	0.1178	0.4117
IND	0.0017	0.0002	0.0029	0.0363	0.0013	0.0915	0.6889	0.0193	0.0016	0.0753	0.0811	0.3111
IT	0.0001	0.0002	0.0009	0.0287	0.0005	0.0783	0.0116	0.7783	0.0001	0.0718	0.0294	0.2217
MAT	0.0002	0.0002	0.000	0.005	0.000	0.0039	0.0018	0.0001	0.9846	0.0029	0.0013	0.0154
RE	0.0004	0.0002	0.0114	0.041	0.0045	0.1901	0.0252	0.0411	0.001	0.5618	0.1232	0.4382
UTI	0.0003	0.0008	0.0087	0.0714	0.0061	0.1978	0.0424	0.0255	0.0011	0.18	0.4658	0.5342
TO	0.0031	0.003	0.0416	0.2515	0.0159	0.6891	0.1407	0.1732	0.007	0.6618	0.5023	*TSI*
TO(own)	0.9934	0.9949	0.9041	0.9334	0.9324	1.2774	0.8297	0.9515	0.9915	1.2236	0.968	0.2263
NET	-0.0066	-0.0051	-0.0959	-0.0666	-0.0676	0.2774	-0.1703	-0.0485	-0.0085	0.2236	-0.032	

Tables 3 and 4 show the liquidity spillovers among all sectors during the GFC and the COVID-19 pandemic periods, respectively. Several notable features are presented in the tables. First, the TSI during the GFC (80.04%) was higher than that during the COVID-19 period (74.46%). The TSI represents liquidity transmissions between all sectors, so we can infer that a higher TSI shows a stronger interdependence of liquidity among sectors during the GFC. Second, MAT showed the highest net spillover during the GFC period, accounting for 23.42%, whereas IT had the largest net spillover at 60.36% during the COVID-19 period. Third, regarding the GFC, the least net spillover was shown in FIN at -29.10%. In contrast, FIN had the third highest percentage (37.83%) during the COVID-19 pandemic, following IT and CD. Lastly, during the COVID-19 pandemic, IND had the lowest net spillover at -59.02%, while the net spillover in IND during the GFC was the third largest at 18.64%, after MAT and IT.

**Table 3:** Summary of liquidity spillovers in all the sectors during the GFC (from August 2007 to March 2009). The optimal lag order according to the AIC is 6.

	CS	CD	CST	ENG	FIN	HC	IND	IT	MAT	RE	UTI	FROM
CS	0.2404	0.0988	0.0403	0.0811	0.0436	0.0487	0.1014	0.1033	0.0907	0.1016	0.0501	0.7596
CD	0.1083	0.2108	0.0483	0.0657	0.0519	0.0542	0.1005	0.1067	0.1094	0.0965	0.0477	0.7892
CST	0.0539	0.0554	0.213	0.0894	0.0288	0.0451	0.1095	0.1076	0.115	0.0713	0.1111	0.787
ENG	0.0917	0.0677	0.0736	0.188	0.0435	0.062	0.0994	0.1026	0.1145	0.0735	0.0834	0.812
FIN	0.0896	0.0713	0.0408	0.0786	0.2451	0.0491	0.0946	0.1065	0.0984	0.0682	0.0578	0.7549
HC	0.1114	0.085	0.0519	0.0862	0.0501	0.2268	0.0791	0.089	0.0917	0.0723	0.0565	0.7732
IND	0.0961	0.0867	0.0717	0.0846	0.0565	0.0452	0.1535	0.1214	0.1217	0.101	0.0616	0.8465
IT	0.0986	0.0918	0.0716	0.0855	0.053	0.046	0.1189	0.1598	0.1218	0.0919	0.061	0.8402
MAT	0.096	0.0917	0.0734	0.0911	0.0494	0.0508	0.1171	0.1181	0.1516	0.0932	0.0674	0.8484
RE	0.1047	0.0904	0.0588	0.0733	0.0441	0.0473	0.1189	0.1097	0.1155	0.1865	0.0507	0.8135
UTI	0.0758	0.0552	0.1097	0.1017	0.0429	0.0492	0.0934	0.0875	0.1039	0.0603	0.2204	0.7796
TO	0.9261	0.794	0.6401	0.8373	0.4639	0.4978	1.0329	1.0525	1.0825	0.8299	0.6471	*TSI*
TO(own)	1.1665	1.0048	0.8531	1.0253	0.709	0.7245	1.1864	1.2123	1.2342	1.0164	0.8675	0.8004
NET	0.1665	0.0048	-0.1469	0.0253	-0.291	-0.2755	0.1864	0.2123	0.2342	0.0164	-0.1325	

**Table 4:** Summary of liquidity spillovers in all the sectors during the COVID-19 pandemic period (from January 2020 to March 2022). The optimal lag order according to the AIC is 5.

	CS	CD	CST	ENG	FIN	HC	IND	IT	MAT	RE	UTI	FROM
CS	0.2476	0.1428	0.0212	0.0603	0.1358	0.0267	0.0057	0.1347	0.1222	0.074	0.0292	0.7524
CD	0.0624	0.207	0.0275	0.0741	0.1328	0.0375	0.0112	0.1687	0.1297	0.0986	0.0505	0.793
CST	0.0379	0.1033	0.3809	0.0527	0.0918	0.0243	0.0023	0.1097	0.0933	0.0632	0.0406	0.6191
ENG	0.0494	0.1257	0.0242	0.2368	0.1468	0.0309	0.0156	0.1261	0.127	0.0909	0.0266	0.7632
FIN	0.0748	0.1529	0.0254	0.0933	0.195	0.0337	0.0073	0.1429	0.1371	0.1021	0.0355	0.805
HC	0.0416	0.125	0.0224	0.0587	0.0994	0.3135	0.003	0.1446	0.0898	0.0631	0.0389	0.6865
IND	0.0219	0.1142	0.0172	0.0632	0.0678	0.0374	0.3405	0.1126	0.0953	0.0844	0.0455	0.6595
IT	0.0682	0.1647	0.0278	0.0682	0.1256	0.0463	0.0085	0.2327	0.121	0.0861	0.0509	0.7673
MAT	0.0704	0.1625	0.0283	0.088	0.1453	0.0332	0.0048	0.154	0.1807	0.0933	0.0394	0.8193
RE	0.0589	0.1542	0.0264	0.0847	0.1442	0.0349	0.0045	0.1412	0.12	0.1781	0.0529	0.8219
UTI	0.036	0.1455	0.0264	0.051	0.0937	0.0289	0.0063	0.1364	0.0883	0.0907	0.2966	0.7034
TO	0.5215	1.3908	0.2469	0.6941	1.1833	0.3336	0.0692	1.3709	1.1237	0.8464	0.41	*TSI*
TO(own)	0.7691	1.5978	0.6278	0.931	1.3783	0.6471	0.4098	1.6036	1.3044	1.0245	0.7066	0.7446
NET	-0.2309	0.5978	-0.3722	-0.069	0.3783	-0.3529	-0.5902	0.6036	0.3044	0.0245	-0.2934	

During the GFC, MAT was the main sector that transmitted total liquidity spillovers to all other sectors, and received total liquidity spillovers from all other sectors at 108.25% and 84.84%. However, FIN had the smallest figures on both occasions, accounting for 46.39% and 75.49%, respectively. During the COVID-19 period, CD was the primary conveyor of liquidity spillovers to other sectors, comprising 139.08%, while RE was the main receiver of total liquidity spillovers from all other sectors at 82.19%. In contrast, the figure for total liquidity spillovers delivered by IND to all other sectors was the smallest, totaling 6.92%, as opposed to CST, which was the least recipient of total liquidity spillovers at 61.91%.

During the GFC, material production and goods trade plunged from 2008 to 2009 across developed economies ([104]). In addition, MAT experienced shocks during the GFC ([50]). Therefore, given the relationship between economic activities and liquidity transmissions, it is safe to conclude that MAT was the main transmitter and recipient of total liquidity spillovers. This is consistent with several studies ([105–107]). Similarly, during the recent COVID-19 pandemic, CD fell more than other sectors, such as CST, at the start of the pandemic in terms of stock performance (Stlouisfed. org, 2021) (https://www.stlouisfed.org/on-the-economy/). Consumer discretionary tends to be susceptible to economic crises ([108–110]), indicating that CD heavily affects other sectors. Accordingly, CD was the foremost contributor to total liquidity spillovers during the COVID-19 pandemic. This is conformable to other studies ([62, 111]). Meanwhile, the COVID-19 shutdown, with the highest degree of uncertainty, resulted in shocks to the real estate sector ([112]). Therefore, we can deduce that RE was the primary recipient of total liquidity spillovers. This is also consistent with previous studies ([112–114]).

### Rolling-window analysis

Here, we present the rolling-window spillover estimates for the average sector liquidity. The estimations are based on 200-day, 30-step windows and horizons. Based on the rolling-window analysis, we can examine how the liquidity relationship between sectors changed during the crises.

**Fig 1:**
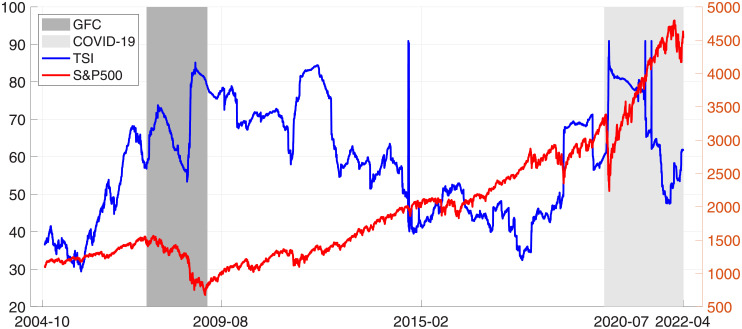
Rolling-windows estimation of the total spillover index and S&P 500 index from October 2004 to March 2022.

Fig 1 shows the rolling window estimation for TSI. Overall, the total spillover index fluctuated wildly during the period from October 2004 to March 2022, while the S&P 500 index showed a steady and considerable increase, except during the GFC and the beginning of the COVID-19 pandemic, when there were sharp falls in S&P 500. Regarding the two crises, the TSI grew drastically during the GFC. However, there was a dip during this period. In contrast, the figure for COVID-19 fluctuated more, with several peaks and lowest points.

Particularly, on October 2, 2014, TSI hit the highest point. There were several reasons for this finding. First, economic growth declined markedly. Second, the first case of Ebola in the US was reported. Third, economic reports were gloomy on October 1. All of these reasons caused investor sentiment to be dented (https://finance.yahoo.com/news/). Meanwhile, amidst the COVID-19 crisis, TSI peaked on March 17 and May 18, 2021. On March 16, the FED started its two-day policy meeting, during which investors expected to obtain more information on inflation and interest rates from the FOMC on March 17. Additionally, retail sales decreased more than expected on March 16. Also, industrial production decreased owing to severe winter storms in February (https://www.nasdaq.com/articles/). Furthermore, on May 18, investors were concerned about increasing inflation and anticipated tight monetary policy. Their sentiment was dampened following a survey by the Federal Reserve, which indicated that nearly a quarter of the adult population was financially poorer from COVID-19 (https://www.nasdaq.com/articles/).

**Fig 2:**
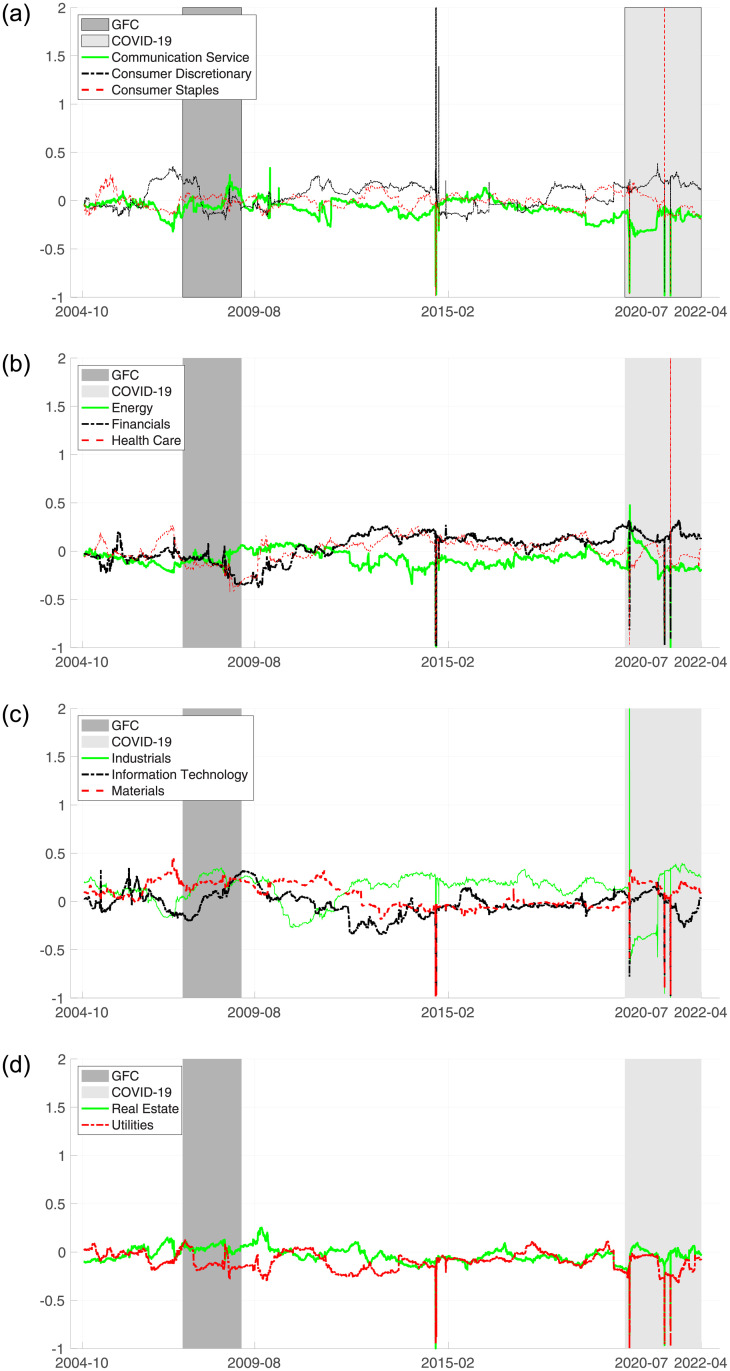
Sector’s dynamic net spillover.

We further scrutinize sectoral net spillovers based on the rolling window analysis in Fig 2. Generally, the net spillover indices for each industry vary over the entire period, displaying several distinct sectors on a particular day. First, during the GFC, net spillovers in all sectors fluctuated suddenly from September 2008 to December 2008. This was attributed to the increased uncertainty and fear of investors due to the US financial crisis (https://www.cnbc.com/). Second, on October 2, 2014, the net spillover index for CD skyrocketed sharply, unlike in other industries. Consumer discretionary tends to be more sensitive to economic cycles than other industries. Therefore, it is safe to conclude that this originated from the poor economic reports on October 1 (https://finance.yahoo.com/news/). Third, the net spillovers for all sectors varied during the COVID-19 pandemic from January 2020 to March 2022. Specifically, on March 20, 2020, IND had the highest net spillover. This is because, at the beginning of COVID-19, the production of capital goods in the US industrial sector was directly affected by production stops and global supply chain disruptions ([115, 116]). On March 17, 2021, the net spillover for CST soared abruptly. Consumer staples are usually impervious to business cycles, as people tend to demand consumer staples at a relatively stable level, that is, in the defensive industry ([62, 117]). However, on March 16, the U.S. Census Bureau announced that retail sales fell above the consensus estimate owing to temporary failure in government aid and unusually bad weather (https://finance.yahoo.com/news/). Hence, it is safe to conclude that CST mainly affected other sectors rather than being affected by others. On May 18, 2021, the figure for HC suddenly hit its top point. The U.S. Food and Drug Administration (FDA) announced actions to tackle the COVID-19 pandemic a day earlier (https://www.fda.gov/news-events/). Therefore, it is safe to conclude that HC became a major influential sector that remarkably affected the liquidity of other sectors on May 18.

## Concluding remarks

This study examines liquidity spillovers among industries in the S&P 500 index using the spillover index proposed by [63] to analyze how liquidity shocks to a specific sector are transmitted across sectors in the US stock market. To this end, we first choose Amihud’s measure ([5]) to calculate liquidity for 11 sectors. Second, by employing the DY model, we compare the total, directional, and net liquidity spillovers for all sectors. Third, we investigate the liquidity spillovers of all and each sector during the GFC and COVID-19 pandemic periods using rolling-window analysis to examine the time-varying trend of sector net spillover as well as total spillover. Furthermore, we clarify our empirical results by comparing them with those of previous studies. In addition, we support our findings by showing that liquidity in financial markets and real economic activities are interrelated. Our main findings and remarks are as follows.

First, the liquidity interconnections between all sectors were reinforced during the GFC and COVID-19 pandemic periods. This finding is consistent with that of other related studies ([118–121]). In particular, this result may have various theoretical backgrounds; among them, the herding effect has convincingly explained the results ([78, 122]).

Second, in accordance with the statistical analysis and total liquidity spillovers of all sectors, HC was the main transmitter of the total liquidity spillovers, whereas UTI received the largest total liquidity spillovers during the entire period. On the one hand, during the GFC, MAT was the primary deliverer and recipient of total liquidity spillovers. On the other hand, during the COVID-19 pandemic period, CD was the biggest conveyor of total liquidity spillovers, and RE was the dominant receiver of total liquidity spillovers. These results are peculiar compared with those of other studies that explained liquidity spillovers between financial markets ([11, 123, 124]).

Third, pursuant to the rolling-window analysis and time-varying net liquidity spillovers of each sector, there were some variations, especially from September 2008 to December 2008, owing to the fear and uncertainty caused by the US financial crisis. Moreover, during the COVID-19 pandemicperiod, certain sectors had distinct net spillover indices. For example, IND, CST and HC reached their highest points in terms of net liquidity spillovers on March 20, 2020, March 17, 2021, and May 18, 2021 respectively. The findings are novel in that previous research addressed volatility spillovers between sectors in financial markets ([125–129]).

Our empirical results have important implications for market participants. First, we notice that the liquidity co-movement between sectors strengthened during the two crises, but sectors transmitting or receiving liquidity shocks differed from crisis to crisis. This shows that the interlinkages among sectors in the US stock market are sensitive to crises, so investors and policymakers should respond to the crisis by understanding the channel of liquidity transmissions.

Second, we reaffirm that liquidity is instrumental in financial markets. Analyzing the US stock market with respect to liquidity, we notice that liquidity shocks to a certain sector are transmitted to other industries, which shows that sudden changes in a specific sector influence market participants’ decisions on where they hold their money. We also perceive the main sectors transmitting and receiving liquidity spillovers in each crisis. Thus, we comprehend the route of liquidity provision and the relationship between liquidity and the US real economy.

Third, our findings can help market participants, such as policymakers and portfolio managers, improve their economic policies and portfolio management strategies. Policymakers can understand the overall mechanism behind liquidity spillovers and implement appropriate measures to correct liquidity imbalances across industries. Given that an event in a local sector may cause risk and return of national stock markets and further influence global financial markets, portfolio managers can also select and manage suitable portfolios by comprehending the degree of liquidity co-movement among sectors and stock markets.

In conclusion, this study has some limitations. First, we use only one liquidity measure, as suggested by [5]. Therefore, we recommend employing other liquidity measures, such as volume-based, price-based, and bid/ask spread-based measures, to comprehensively analyze liquidity spillovers. Such a future study will help us better understand the liquidity relationships between sectors and likely support our findings. Second, we suggest extending our analysis to other stock markets, such as the European and Asian stock markets. As the COVID-19 pandemic has affected all countries, it is important to investigate liquidity spillovers in other stock markets. Future studies will allow us identify the similarities or dissimilarities between the empirical findings of the suggested studies and those of our study. Third, we propose a liquidity spillover analysis in terms of portfolio management because we only explore the sectoral co-movement of liquidity in the US stock market. Further research is needed to analyze this to help market participants improve their strategies.
